# Cesarean section may increase the risk of both overweight and obesity in preschool children

**DOI:** 10.1186/s12884-016-1131-5

**Published:** 2016-11-03

**Authors:** Erigene Rutayisire, Xiaoyan Wu, Kun Huang, Shuman Tao, Yunxiao Chen, Fangbiao Tao

**Affiliations:** 1 Department of Maternal, Child & Adolescent Health, School of Public Health, Anhui Medical University, 81 Meishan Road, Hefei, Anhui Province 230032 China; 2 Anhui Provincial Key Laboratory of Population Health & Aristogenics, Hefei, 230032 China

**Keywords:** Elective cesarean section, Non-elective cesarean section, Oveweight, Obesity, Preschool children

## Abstract

**Background:**

The increase rates of cesarean section (CS) occurred at the same period as the dramatic increase of childhood overweight/obesity. In China, cesarean section rates have exponentially increased in the last 20 years and they now exceed World Health Organization (WHO) recommendation. Such high rates demand an understanding to the long-term consequences on child health. We aim to examine the association between CS and risk of overweight and obesity among preschool children.

**Method:**

We recruited 9103 children from 35 kindergartens in 4 cities located in East China. Children anthropometric measurements were taken in person by trained personnel. The mode of delivery was classified as vaginal or CS, in sub-analyses we divided cesarean delivery into elective or non-elective. The mode of delivery and other parental information were self-reported by parents. Multivariate logistic regression analysis was used to examine the associations.

**Results:**

In our cross-sectional study of 8900 preschool children aged 3–6 years, 67.3 % were born via CS, of whom 15.7 % were obese. Cesarean delivery was significantly associated with the risk of overweight [OR 1.24; (95 % CI 1.07–1.44); *p* = 0.003], and the risk of obesity [OR 1.29; (95 % CI 1.13–1.49); *p* < 0.001] in preschool children. After adjusted for child characteristics, parental factors and family income, the odd of overweight was 1.35 and of obesity was 1.25 in children delivered by elective CS.

**Conclusion:**

The associations between CS and overweight/obesity in preschool children are influenced by potential confounders. Both children delivered by elective or non-elective CS are at increased risk of overweight/obesity. Potential consequences of CS on the health of the children should be discussed among both health care professionals and childbearing women.

## Background

Globally, cesarean section (CS) rate increased from 12 % in 2000 to 15.5 % in 2012 [1]. In China, CS rate is nearly four times compared to the World Health Organization (WHO) recommendation. To be noted is that previously the rates of birth by CS in China had increased from 18 % in 1990–1992 to 40 % in 2000 and exceed 50 % in some large cities [2]. In 2012, cesarean section rate was 54 % among all deliveries in rural areas of China [3] while in the large cities such as Beijing CS rates have reached 58.5 % in 2015 [4]. The increased rates of cesarean section occurred at the same period as the dramatic increase of childhood overweight/obesity. Annual increase of overweight/obesity in children is about 0.5–0.7 % in United States of America (USA), Australian, United Kingdom (UK) and China [5]. In China, the prevalence of childhood overweight and/or obesity increased from 12.6 % in 1997 to 22.1 % in 2009 [6]. In 2014, 26.2 % of children in the coastal province of China were obese [7].

Birth by CS has been linked with the risk of overweight or obesity during childhood, the association between these two is uncertain. Regarding the association between CS and childhood obesity, recent studies including a meta-analysis observed that cesarean section is associated with increased risk of childhood overweight/obesity [8, 9]. In contrast, few studies reported no significant association between CS and overweight/obesity in children at the age of 6 or 7 [10, 11]. These conflicting results may be partly explained by different adjusted confounders; mainly the age at which the outcome (overweight or obesity) were assessed, and may also be due to not distinguished elective and non-elective CS.

Few studies in China have examined the association between cesarean section and childhood overweight/obesity, and the findings have not been conclusive [12, 13]. None of these studies examined the outcomes (overweight and obesity) separately; this may result in high or low risk estimates than examining the risk in each group. Previous studies that included maternal weight in full adjustment reported lower odds of obesity or overweight among children delivered by CS. Therefore, we hypothesized that including pre-pregnancy BMI and paternal factors in full adjustments model may reduce the odds of both overweight and obesity among the preschool children delivered by CS. In addition, the discrimination between elective and non-elective CS might have different effects on childhood overweight or obesity. Given the previous conflicting results, we sought to further examine the association between cesarean section and risk of childhood overweight and obesity in large sample of preschool children as well as examine this risk in subtypes of CS (elective and non-elective CS). Additionally, we also examined these associations after controlling potential confounders such as birth status, pre-pregnancy BMI and other parental characteristics.

## Methods

### Study population

**Fig. 1:**
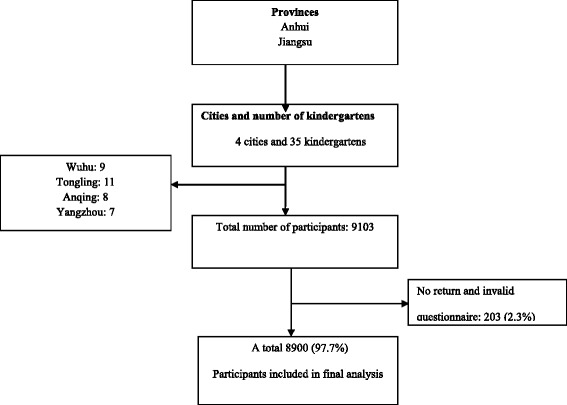
Flow chat diagram for study population

The study population was recruited from March to June 2015 and the cluster sampling technique was used to select 35 kindergartens from 4 cities: Wuhu, Tongling, Anqing and Yangzhou located in Eastern China. After completing anthropometric measures of 9103 children aged 3–6 years, questionnaires were taken home by children to be completed by parents, 8900 of 9103 were returned and valid (Fig. 1).

### Outcome variables

Data on weight and height for all children were collected in person by trained personnel at each selected kindergarten. Children were requested to wear light clothing and stand erect, barefoot, and at ease while being measured. Weight was recorded to the nearest 0.1 kg with standardized scale and height to the nearest 0.1 cm with a portable stadiometer. Both scales and stadiometers were calibrated before use. The average of two height measures was used; in case the two differed by greater than 0.5 cm, a third measurement was taken and the average of the two closest was used in all analyses. Overweight and obesity were defined according to WHO Child Growth Standards for age- and sex- specific cut-off points [14, 15]. Overweight was defined as a BMI between 85^th^ and < 95^th^ percentile, and obesity was defined as a BMI ≥ 95^th^ percentile for age and sex.

### Definition of delivery mode

Delivery mode was determined by asking parents the following question: ‘Which type of delivery did the mother have to give birth to this child?’ Firstly, they were two options: vaginal delivery or cesarean section. Additionally, participants who responded CS were asked to specify if it was elective or non-elective.

### Potential confounders

Information about maternal factors was collected through a self-report questionnaire taken home by children. Parents were asked to complete questionnaires and return them within a period of two weeks. The information requested included maternal age in years, maternal education level (whether the mother completed primary school, middle school, high school, college or above), maternal smoking (never, one or less cigarette per day, 1–5 cigarettes per day, 6 or more per day) maternal drinking (never, 1–2 drinks per day, ≥3 drinks per day), pre-pregnancy weight in kg, maternal weight before delivery in kg, breastfeeding status (never, less than 4 months, 4–5 months, 6 month and above). Child related factors including birth weight in grams, age, gestational age (preterm/full-term), gender (male/female) were also recorded. Paternal factors including age, education level, smoking, drinking, height and weight were as well collected and information about family monthly income was completed by parents by use of the same questionnaire.

Maternal pre-pregnancy BMI was calculated based on self-reported maternal height and pre-pregnancy weigh. We calculated gestational weight gain as the difference between self-reported weight at the last prenatal care and self reported pre-pregnancy weight (mothers were reminded to check the weight on their antenatal and postnatal care card). The fathers’ BMI was calculated from self-reported height and weight. Parental body mass index (BMI) was calculated as weight (kg)/ [height (m)] ^2^) and categorized as normal (less than 25.0), overweight (25.0–29.9) and obese (30.0 or more).

### Statistical analysis

Children and parental characteristics were measured by using Chi-Square test for categorical characteristics and *t* test for continuous characteristics in relation to delivery mode. Categorical variables were presented as frequency with percentage while continuous variables were expressed as the mean ± standard deviation. In our analysis, we first dichotomized delivery mode as cesarean section and vaginal delivery. Multinomial logistic regression was used to assess the associations of CS and overweight or obesity. In supplementary analyses we classified delivery mode in three categories: Elective CS, non-elective CS and vaginal delivery, and then examined the relationship between elective or non-elective CS and overweight or obesity, in all analyses normal BMI were used as the control group.

Multinomial logistic regression was applied to estimate the unadjusted and adjusted ORs (95 % CI) for overweight and obesity. The multivariable model was adjusted for: model 1 (child factors), model 2 (model 1+ maternal factor), model 3 (child factors + maternal factors + paternal factors and family income), to evaluate association between CS and overweight, or obesity in preschool children. *P* values less than 0.05 were considered to be statistically significant. All analysis was performed using SPSS, Version 10 (SPSS Inc, Chicago, IL, USA).

## Results

Child and parental characteristics by delivery mode are presented in Table 1. Of 8900 preschool children, 67.3 % were born by cesarean section. As our results have it, mothers who delivered after the age of 36, and who were overweight or obese before pregnancy, and with a relatively higher education level, and gained more weight during pregnancy presented the risk of delivery by cesarean section (*P* < 0.05). A total of 939 (15.7 %) children delivered by cesarean section were obese, 25.2 % of children delivered by CS were not breastfed. Gestational age, birth weight, gender, maternal smoking and maternal drinking were not associated with the mode of delivery. Out of 5993 children delivered by CS, 4016 (67 %) were non-elective, and 1977 (33 %) were elective CS. Paternal age was associated with the delivery mode (*P* < 0.001). In addition, fathers’ BMI, education level, smoking, and family income were associated with the mode of delivery (*P* < 0.05).

**Table 1:** Children and parental characteristics in relation to delivery mode in 2015

Characteristics	Vaginal delivery	Cesarean section	*p* value
N (%)	2907 (32.7)	5993 (67.3)	
Maternal factors			
Maternal age			
19–24	51 (1.8)	38 (0.7)	<0.001
25–29	859 (29.5)	1501 (25.0)	
30–35	1604 (55.2)	3433 (57.3)	
36 or older	393 (13.5)	1021 (17.0)	
Maternal education			
Completed primary school	70 (2.4)	87 (1.5)	<0.001
Completed middle school	483 (16.6)	777 (13.0)	
Completed high school	652 (22.4)	1488 (24.8)	
College or above	1702 (58.6)	3641 (60.7)	
Smoking			
Never	2896 (99.6)	5948 (99.2)	0.054
One or less cigarettes/day	6 (0.2)	10 (0.2)	
1–5 cigarettes per day	3 (0.1)	23 (0.4)	
6 or more cigarettes per day	2 (0.1)	12 (0.2)	
Drinking			
Never	2863 (98.5)	5872 (98.0)	0.240
Occasionally	40 (1.4)	112 (1.8)	
Daily	4 (0.1)	9 (.2)	
Pre-pregnancy BMI			
Normal	2690 (92.9)	5363 (89.9)	<0.001
Overweight	193 (6.7)	572 (9.6)	
Obesity	12 (0.4)	30 (0.5)	
Gestational Weight Gain (kg), mean ± SD	14.10 ± 7.52	15.78 ± 8.07	<0.001
Child factors			
Birth weight			
Normal	2811 (98.7)	5814 (97.0)	0.420
Low birth weight	96 (3.3)	179 (3.0)	
Gestational age			
Preterm	169 (5.8)	312 (5.2)	0.235
Full-term	2738 (94.2)	5681 (94.8)	
Gender			
Male	1529 (52.6)	3181 (53.1)	0.670
Female	1378 (47.4)	2812 (46.9)	
Duration of breastfeeding			
Never	587 (20.2)	1509 (25.2)	<0.001
Less than 4mo	600 (20.6)	1209 (20.2)	
4–5 mo	786 (27.1)	1493 (24.9)	
6+	934 (32.1)	1782 (29.7)	
Child BMI (kg/m)			
Normal	2265 (77.9)	4309 (71.9)	<0.001
Overweight	293 (10.1)	745 (12.4)	
Obesity	349 (12.0)	939 (15.7)	
Paternal factors			
Age			
19–24	12 (0.4)	12 (0.2)	<0.001
25–29	409 (14.1)	623 (10.4)	
30–35	1574 (54.1)	3301 (55.1)	
36 or older	912 (31.4)	2057 (34.3)	
Education level			
Completed primary school	32 (1.1)	40 (0.7)	<0.05
Completed middle school	1029 (35.4)	1957 (32.7)	
Completed high school	720 (24.8)	1648 (27.5)	
College or above	1126 (38.7)	2348 (39.1)	
Smoking			
Never	1408 (48.4)	2662 (44.4)	<0.05
One or less cigarettes/day	159 (5.5)	331 (5.5)	
1–5 cigarettes per day	528 (18.2)	1233 (20.6)	
6 or more cigarettes per day	812 (27.9)	1767 (29.5)	
Drinking			
Never	1692 (58.2)	3348 (55.9)	0.111
Occasionally	948 (32.6)	2057 (34.3)	
Daily	267 (9.2)	588 (9.8)	
Father BMI			
Normal	1871 (65.1)	3634 (61.2)	<0.05
Overweight	898 (31.2)	2048 (34.5)	
Obesity	107 (3.7)	256 (4.3)	
Family income			
Lower income	364 (12.5)	642 (10.7)	<0.05
Middle income	2220 (76.4)	4693 (78.3)	
Higher income	323 (11.1)	658 (11.0)	

*BMI* body mass index, *SD* standard deviation, *Mo* month

The odd ratio (OR) of overweight and obesity in preschool children aged 3–6 years with association of delivery mode were estimated using logistic regression (Table 2). In the unadjusted analysis, cesarean section was significantly associated with overweight [OR 1.33; (95 % CI 1.15–1.54)] and with obesity [OR 1.41; (95 % CI 1.23–1.61)], for both *p* < 0.001. Adjusted for child factors (Model 1) did not notably alter our results. The estimates remained statistically significant for overweight [OR 1.27; (95 % CI 1.09–1.47)], *p* < 0.05 and for obesity [OR 1.32; (95 % CI 1.15–1.52)], *p* < 0.001 in model 2. In our full adjustment we include paternal factors and family income to the previous models birth by cesarean section remained significantly associated with both overweight and obesity., However, in full model the odds ratio decreased slightly for overweight [OR 1.24; (95 % CI 1.07–1.44)], *p* < 0.05 and for obesity [OR 1.29; (95 % CI 1.13–1.49)], *p* < 0.001.

**Table 2:** Unadjusted and adjusted odds ratios or overweight and obesity among the pre-school children by Delivery Mode in 2015

	Overweight	*p* value	Obesity	*p* value
Unadjusted				
Vaginal delivery	1.0		1.0	
Cesarean section	1.33 (1.15–1.54)	<0.001	1.41 (1.23–1.61)	<0.001
Elective CS	1.44 (1.20–1.72)	<0.001	1.34 (1.13–1.59)	0.001
Non-elective CS	1.28 (1.09–1.49)	0.002	1.44 (1.25–1.66)	<0.001
Model 1				
Vaginal delivery	1.0		1.0	
Cesarean section	1.33 (1.15–1.53)	<0.001	1.40 (1.22–1.61)	<0.001
Elective CS	1.43 (1.19–1.71)	<0.001	1.32 (1.11–1.57)	0.001
Non-elective CS	1.28 (1.09–1.49)	0.002	1.44 (1.25–1.66)	<0.001
Model 2				
Vaginal delivery	1.0		1.0	
Cesarean section	1.27 (1.09–1.47)	0.001	1.32 (1.15–1.52)	<0.001
Elective CS	1.36 (1.13–1–64)	0.001	1.27 (1.06–1.51)	0.007
Non-elective CS	1.22 (1.04–1.43)	0.011	1.35 (1.16–1.56)	<0.001
Model 3				
Vaginal delivery	1.0		1.0	
Cesarean section	1.24 (1.07–1.44)	0.003	1.29 (1.13–1.49)	<0.001
Elective CS	1.35 (1.12–1.63)	0.001	1.25 (1.05–1.49)	0.012
Non-elective CS	1.19 (1.01–1.40)	0.029	1.32 (1.13–1.53)	<0.001

Model 1: Adjusted for child factors: Gender, age, birth weight, gestational age, duration of breastfeeding

Model 2: Model 1 + Adjusted for maternal factors: gestational weight gain, smoking, drinking, maternal age, pre-pregnancy BMI, education level

Model 3: Model 1 + Model + 2 Adjusted for paternal factors and family income: age, education, smoking, drinking, BMI, family income

In addition, we distinguish elective and non-elective CS to analyze the risk of each on overweight and obesity in preschool children. Unexpectedly, in unadjusted or adjusted analyses, significantly increased odds of overweight and obesity were observed in both elective and non-elective CS. In unadjusted analysis, the odds ratio of overweight and obesity were [OR 1.44; 95 % (1.20–1.72)] and [OR 1.34; (1.13–1.59)] respectively, in preschool children delivered by elective CS. The estimates remained significant for both overweight [OR 1.35; (1.12–1.63)] and obesity [OR 1.25; (1.05–1.49)] in the fully adjusted model.

## Discussion

In this cross-section study, we found a positive association between birth by cesarean section and childhood overweight and obesity. We found a 24 % increase risk of overweight and a 29 % increase risk of obesity in preschool children delivered by cesarean section. Furthermore, the significant increased risk of overweight and obesity was observed in both children delivered by elective or non-elective CS. Our results suggested that the risk of overweight and obesity among children born via CS decreased when controlling potential confounders. Our results are consistent with several previous studies [9, 13, 16] but in contrast with some [10].

A greater risk of obesity with significant association has been reported in children delivered by CS compared with those who were delivered vaginally. In 2014, Salehi-Abargouei et al. [17] reported statistically significant risk of general obesity (OR: 2.46 and 95 % CI 1.30–4.63 in children 6–12 years). Rooney et al. [18] reported statistically significant risk of childhood obesity (RR: 2.94 and 95 % CI 1.36–6.34 in children 4–5 years). Muller et al. [19], in 2015 reported a 46 % higher risk of obesity in 7-year old children delivered by CS. However, these studies did not distinguish overweight and obesity. Another drawback is that they had small sample sizes and other methodological aspects might have contributed to higher risk of obesity observed in children delivered by cesarean section. Chinese birth cohort indicated a moderate risk of being overweight in children delivered by CS (OR: 1.13 and 95 % CI (1.08–1.18) in children 3–7 years) [12]. Like many other previous studies, the risk for overweight and obesity in children delivered by CS was not estimated separately in Chinese birth cohort.

In line with our findings, a recent Peruvian prospective cohort which distinguished overweight and obesity reported significant associations between CS and risk of childhood overweight (RR: 1.51; 95 % CI: 0.98–2.35) and obesity (RR: 2.25; 95 % CI: 1.36–3.74) among children with mean age of 5.3+ 0.4 years [9]. Among USA children in grade six, Wang L. et al. [20] found increased risk of overweight (OR: 1.86; 95 % CI: 1.27–2.73) and obesity (OR: 1.87; 95 % CI: 1.19–2,95) in children delivered by CS compared to those delivered vaginally. In contrast, few studies reported non-significant association but elevated higher odds of obesity among children delivered by CS compared to children delivered vaginally at the age of 2 [21], and at the age of 6 [10]. Indeed, the discrepancy in these results may be explained by the age difference the outcome (overweight and/or obesity) was assessed among the study population.

In the present study, we found a 29 % increased risk of obesity in preschool children delivered by CS aged 3–6 years. In line with our findings, a recent meta-analysis studied the association between cesarean section and childhood obesity, reported a 34 % increased risk of obesity in children aged 2-18 years delivered by CS [8]. Similarly, Pei et al. [10] found a significant association between CS and obesity in children at the age of 2 years. In addition, we found significant association between cesarean section and being overweight in preschool children aged 3–6. In contrast, Pei et al. [10] reported no significant associations between cesarean delivery and overweight at age 2, 6 and 10.

Unexpectedly, we found that both elective and non-elective CS increase the risk of overweight and obesity in preschool children. We observed a significant association and a 35 % increased risk of overweight in preschool children delivered by elective CS compared with those delivered vaginally. Several previous studies investigated the association between CS and obesity did not discriminate between elective and non-elective CS. A recent Chinese birth cohort study reported 18 % increased risk of overweight among the infants delivered by maternal request CS compared to vaginally delivered infants [12]. In our study, the increased risk of overweight and obesity in children delivered by non-elective CS may be linked to medically indicated CS performed due to the pregnancy complications, this occurred prior to labor and rupture of the membranes and deprived the exposure to vaginal microbiota. With the dramatic increase in the rate of CS today, potential consequences of CS on the health of the children should be discussed among both health care professionals and childbearing women, and introducing primary maternity care should be a top priority.

It has been speculated that lack of exposure to maternal gut microbiota for children born via CS could be a potential biological mechanism for development of obesity in early or later childhood [22], however the exact mechanisms of this remains to be elucidated [23]. Regardless of delivery mode, Karlsson et al. [24] confirmed the differences in gut microbiota between preschool children with overweight/obesity and those with normal BMI. Specifically, a recent review reported that during their first 3 months of life, infants delivered by CS had significantly lower Bifidobacterium and Bacteroides genera compared with vaginally delivered infants [25]. Moreover, Ley et al. [26] suggested that change in the gut microbiota may explain the effect of delivery mode on the development of obesity in children.

In fact, the acquisition of early microbiota was found to be influenced by delivery mode. Colonization by gut microbiota dominated by Bifidobacterium and Collinsella species delayed by 6 months in infants delivered by cesarean section, and this might predict adiposity at 18 months of age and later risk of childhood obesity [27]. Kallimaiki et al. [28] observed that children who became overweight at the age of 7 had a lower quantity of bifidobacteria at 6 months and 1 year of age than those whose weight remained normal. The disruption of the gut microbiota in early infancy may explain the growing number of childhood diseases including obesity.

In our study, after including pre-pregnancy BMI, and socioeconomic status as potential confounders; the significant association between CS and childhood overweight and obesity persisted. The findings of our study were in accordance with other studies which controlled maternal BMI [16] and socioeconomic status [17, 29].

Regardless the mode of delivery, a few studies found a significant association between paternal BMI and childhood obesity at age 4.5. [30]. Given the importance of paternal BMI on the development of childhood obesity, our study suggested paternal BMI as another potential confounder to study the association between delivery mode and childhood obesity. The evidence that children from obese parents are more likely to become obese at preschool age highlights the role of genetic factor in development of obesity.

The major strength of this cross-section study was a large sample size, in person measurement of children body size, potential confounders including maternal BMI, gestational weight gain, and duration of breastfeeding, birth weight, paternal BMI, and socioeconomic status. The current study classified CS into elective or non-elective, which was a limitation in several previous studies. In addition, adjusting for several models to detect any changes in odds of overweight and obesity in relation to delivery mode is also one of the strengths of this study. Delivery mode, self reported pre-pregnancy and before delivery weight reported by parents were assumed to be accurate. Like other retrospective study, this study has some limitations that may include recall bias for some variables that we adjusted for in multiple regression models. Recall bias of some confounders may have the effect on the risk estimate. Conducting this study in China, a country with higher national prevalence of cesarean section may increase the chance of detecting association between any type of CS and overweight and obesity in preschool children population. In addition, our study is lacking information on antibiotics use during pregnancy as potential confounder; it has been observed that maternal exposure to antibiotics in second or third trimester of pregnancy increased the risk of childhood obesity at age 7 years [19]. However, this limitation is shared with other studies which reported significant association between delivery mode and overweight or obesity [9, 17, 20].

## Conclusions

Our cross-sectional study found significant increased risk of both overweight and obesity in preschool children delivered by cesarean section; these associations were observed in both children delivered by elective and non-elective CS compared to those who were delivered vaginally. The odds of overweight and obesity decreased after controlling potential confounders. Further studies on these associations are warranted to shed the light on the remaining uncertainty and should focus on the potential biological mechanism.

## Abbreaviations

BMI: Body mass index
CI: Confidance Interval
CS: Cesarean section
OR: Odd ratio
